# A leakage-aware entropy screening protocol for structured biomarker panel evaluation in ovarian cancer risk modelling^✰^^✰✰^

**DOI:** 10.1016/j.mex.2026.103880

**Published:** 2026-03-21

**Authors:** DSS Lakshmi Kumari P, Maragathavalli P

**Affiliations:** a IT, Puducherry Technological University, Puducherry, India; b IT, SRKR Engineering college, Bhimavaram, Andhra Pradesh, India

**Keywords:** Ovarian cancer, Biomarker panels, Von neumann entropy, Data leakage mitigation, Information-theoretic screening, Gradient boosting

## Abstract

In biomedical risk modeling, entropy-based feature screening is being increasingly used, but covariance statistics have been commonly calculated based on entire datasets before validation. It may introduce a small amount of information leakage and spur a perceived panel synergy especially when administrative or post-outcome variables are present.

This article presents a leakage-aware entropy screening protocol for structured biomarker panel evaluation. The workflow integrates (i) systematic administrative variable auditing, (ii) fold-restricted von Neumann entropy estimation derived from covariance-based density matrices, and (iii) cross-validated predictive benchmarking within a unified implementation framework. Entropy computation is strictly confined to training partitions under stratified cross-validation, and panel definitions are fixed prior to analysis to avoid grouping bias.

The protocol is demonstrated using a structured ovarian cancer dataset from the PLCO Cancer Screening Trial to illustrate leakage-controlled panel-level entropy estimation and validation-consistent benchmarking. The workflow provides an auditable and reproducible framework for multivariate panel screening and is adaptable to other structured biomedical datasets requiring grouped feature evaluation.

## Graphical abstract

Workflow for leakage-aware entropy screening in biomarker panel evaluation. Administrative variables that may encode outcome information are audited and removed before entropy estimation. Von Neumann entropy is computed from fold-restricted covariance matrices within stratified cross-validation to prevent test-fold contamination. Panel-level entropy is coupled with cross-validated gradient-boosting performance to provide a validation-consistent assessment of panel complexity, turning entropy screening into a reproducible, leakage-controlled evaluation framework for structured biomedical datasets.

**Figure:**
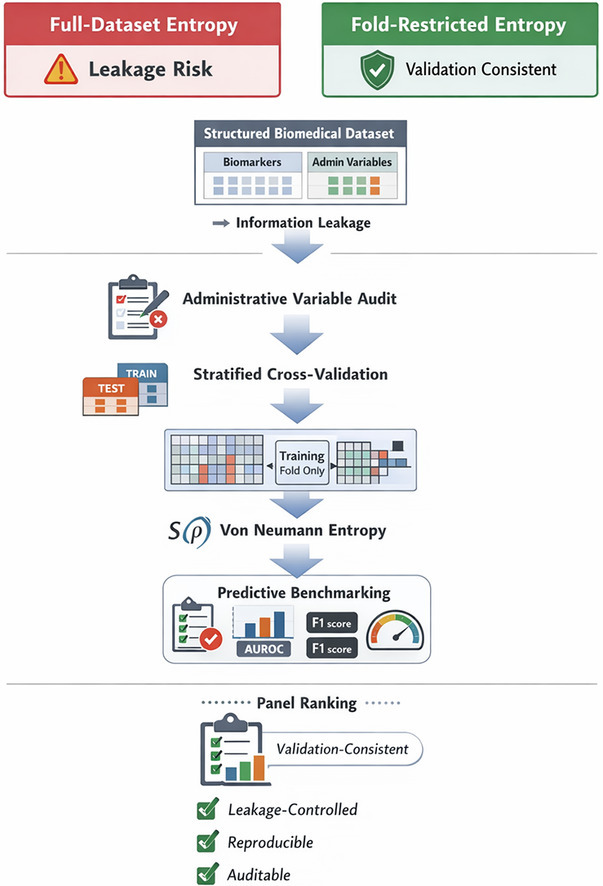

## Method summary


 

1. Formal auditing and removal of administrative variables prior to estimation of entropy.
2. Fold-restricted covariance-based von Neumann entropy computation.
3. Integrated prediction and entropy evaluation in cross-validation.

## Specifications table

**Table:** 

**Subject area**	Computer Science
**More specific subject area**	Computer Vision, Deep learning, Biomarker panel screening and entropy based multivariate analysis
**Name of your method**	Leakage-aware quantum-inspired entropy screening
**Name and reference of original method**	Von Neumann entropy from quantum information theory (Nielsen & Chuang, 2011) adapted to covariance-derived density matrices
**Resource availability**	https://github.com/divyapannasa/-Quantum-Inspired-Entropy-Mapping-for-Ovarian-Cancer-Risk-Modelling

## Background

Ovarian cancer remains one of the most lethal gynecological malignancies due to late-stage detection and heterogeneous biological presentation [1,2]. Recent studies have applied many machine learning approaches for risk stratification by using serum biomarkers, imaging features, and clinical covariates [3,4]. Although these models demonstrate moderate predictive performance, moreover, most of them evaluate biomarkers either individually or through aggregate modeling without systematically assessing redundancy within grouped panels.

In an earlier review by an author [5] in ovarian cancer machine learning approaches, we observed that performance improvements are often reported without detailed examination of feature interaction structure. Subsequently, an author [6] has done a comparative analysis of classification frameworks that further highlighted that feature selection and data balance significantly influence the accuracy reported. However, neither study explicitly quantified multivariate information structure at the panel level.

In addition, Information-theoretic approaches provided a principled alternative framework for structured feature group evaluation. Mutual information has been widely used for feature selection [7], and higher-order interaction decomposition frameworks have been proposed to distinguish redundancy from synergy [8]. Von Neumann entropy (vNE), originally defined in quantum information theory [9,10], has been adapted to analyze covariance-derived density matrices in complex biological systems [11]. The vNE captures eigenvalue dispersion of multivariate covariance structure, enabling panel-level complexity assessment compared with the classical Shannon entropy applied to univariate distributions.

In recent studies the authors [12,13] identified that predictive modeling in biomedical datasets is particularly vulnerable to subtle data leakage, especially when administrative or follow-up variables encode outcome information. The leakage can absolutely increase both entropy-derived metrics and predictive performance, that leads for the miss interpretations of interaction effects.

Preliminary exploratory entropy mapping revealed sensitivity of panel synergy estimates to administrative variables. This observation motivated formalization of a leakage-aware protocol. The objective is to provide a reproducible framework for panel-level screening prior to biological interpretation or downstream modeling.

Conventional leakage-aware machine learning practice emphasizes that feature selection, hyperparameter tuning, and preprocessing must be performed within the training portion of each validation split to avoid optimistic bias [14,15]. However, most such discussions are framed for individual-feature ranking or model tuning rather than for group-level information measures derived from covariance structure. In entropy-based biomarker panel screening, leakage can arise not only through standard preprocessing steps but also through estimation of the covariance matrix itself if panel entropy is computed on the full dataset before validation. The present workflow addresses this gap by restricting entropy computation to training folds, integrating structured administrative-variable auditing, and coupling entropy screening with cross-validated predictive benchmarking.

## Method details

Entropy-based screening methods are increasingly used to assess multivariate structure in biomedical datasets. However, in many practical implementations, entropy or covariance statistics are computed using the entire dataset prior to model validation. While this simplifies computation, it creates a subtle but important methodological issue: covariance structure derived from all samples may indirectly incorporate information from test partitions. In predictive modelling settings, this can lead to overly optimistic interpretations of panel complexity and interaction strength.

During our exploratory entropy mapping of ovarian cancer biomarker panels, certain panel combinations appeared to demonstrate moderate synergy. Upon closer inspection, we observed that administrative and follow-up variables were contributing disproportionately to covariance structure. Once these variables were removed and entropy was recomputed under fold-restricted conditions, the apparent synergistic effects were attenuated.

This observation motivated the development of a leakage-aware entropy screening protocol. The purpose of this methodology is not to introduce a new classifier, but to establish a structured and auditable workflow for panel-level entropy evaluation under strict validation constraints.

The methodological contributions of the present framework are:

1. Administrative variable audit
2. Predefined panel grouping
3. Fold-restricted preprocessing and entropy computation
4. Coupled predictive benchmarking under identical validation splits

These elements collectively address an under-documented gap in entropy-based screening workflows, Each component is described below.

### Dataset and administrative variable audit

The sources used were the Prostate, Lung, Colorectal, and Ovarian (PLCO) Cancer Screening Trial (*n* = 1101 samples after filtering).

Prior to any computation of entropy or predictive modelling, a systematic leakage audit was performed. Administrative indicators that are often found in biomedical datasets are eligibility flags, case resolution codes, and follow-up outcomes. These variables can be explicit or indirectly coded in the status of the outcome. Entropy measures could be manifesting administrative encoding as opposed to inherent biomarker structure should such variables be added to covariance estimation.

The removal of variables was done according to the following criteria:

1. Was mortality or represented exit status, or follow-up endpoint.
2. Coded eligibility or enrolment criteria.
3. Reflective case-control administrative labeling.
4. Outcome-ascertained variables.

Confirmation was done by correlation analysis with the binary outcome, examination of the significance of features in initial gradient boosting, and by examining the description of the variables manually. It was not until this audit was done that entropy analysis was underway.

### Panel definition and grouping strategy

To avoid data-driven grouping bias, features were organized into predefined groups prior to analysis:

1. Serum biomarker panels (Panel A–E)
2. Reproductive variables
3. Clinical-Lifestyle variables
4. All-Biomarkers composite group

Panel definitions were fixed and were not modified based on predictive performance. This ensures that entropy comparisons reflect intrinsic structural properties rather than optimized grouping

### Fold-restricted preprocessing and validation

To avoid optimistic bias arising from inadvertent information transfer across splits, Stratified 5-fold cross-validation (random_state = 42) was employed. For each fold k,Data were partitioned into training ($X_{\text{train}}^{\left(k\right)}$) and test ($X_{\text{test}}^{\left(k\right)}$) subsets. All preprocessing operations were carried out strictly within the training partition. Variables previously flagged during the leakage audit were removed before further analysis. Median imputation statistics were estimated from $X_{\text{train}}^{\left(k\right)}$, followed by estimation of standardization parameters. These fitted transformations were then applied to the corresponding test data without refitting, thereby preserving separation between training-derived statistics and held-out samples.

Entropy estimation was also restricted to the training subset within each fold. Denoting the entropy operator by E, the fold-specific entropy can be expressed as $S_{k}=E\left(X_{\text{train}}^{\left(k\right)}\right)$

Since $X_{\text{test}}^{\left(k\right)}$ is not used in covariance estimation,

$$\frac{\partial S_{k}}{\partial X_{\text{test}}^{\left(k\right)}}=0$$

This ensures that entropy values do not incorporate information from held-out samples.

### Von Neumann entropy for panel-level complexity

For each feature group $G_{j}$ within a training fold, the empirical covariance matrix was computed:

$$C_{j}=\text{Cov}\left(X_{j}\right)$$

To enable comparison across panels of varying dimensionality, a density matrix was constructed via trace normalization:

$$\rho_{j}=\frac{C_{j}}{\text{tr}\left(C_{j}\right)}$$

Trace normalization removes dependence on absolute variance magnitude and instead emphasizes eigenvalue dispersion.

Eigenvalues $\lambda_{k}$ of $\rho_{j}$ were computed, and values below $10^{-12}$ were discarded to ensure numerical stability. Von Neumann entropy was then calculated as:

$$S\left(\rho_{j}\right)=-\sum_{k}\lambda_{k}\text{lo}g_{2}\lambda_{k}$$

Bootstrap resampling (B=1000) was used to estimate confidence intervals for fold-wise entropy estimates.

Unlike classical Shannon entropy applied to univariate distributions, von Neumann entropy captures multivariate covariance structure and reflects dispersion across principal components.

### Panel combination evaluation

To evaluate whether multivariate complexity translates into predictive utility, panel entropy was systematically paired with supervised evaluation.

For each individual panel and each pairwise combination:

1. Fold-restricted entropy was computed.
2. A gradient boosting classifier (XGBoost) was trained within the same cross-validation framework.
3. Performance metrics were recorded:

○ AUROC
○ Accuracy
○ Macro-F1 score
○ Brier score
○ Expected Calibration Error (ECE)

By aligning entropy estimation and predictive benchmarking within identical validation splits, the framework enables direct assessment of whether increased structural complexity corresponds to measurable predictive uplift.

### Computational environment

All analyses were performed using:

• Python 3.10
• NumPy
• SciPy
• Scikit-learn
• XGBoost

Random seed was fixed at 42 to ensure reproducibility.

## Method validation

To illustrate the implementation of the proposed leakage-aware entropy screening protocol, the workflow was executed using the PLCO ovarian cancer dataset. The objective of this demonstration is to show how the protocol operates under leakage-controlled conditions and how entropy estimation behaves when administrative variables and full-dataset covariance contamination are addressed.

Four verification checks were performed to illustrate different components of the workflow:

1. Fold-isolated panel entropy ranking
2. Bootstrapped panel-pair synergy stability
3. Entropy–prediction coupling under consistent cross-validation splits
4. Comparison with naive full-feature modeling to illustrate leakage inflation

Together, these checks demonstrate how the protocol evaluates multivariate panel structure while maintaining strict separation between training and validation data.

### T1. Entropy ranking of fold isolation at panel level

Von Neumann entropy (vNE) was calculated at the panel level (normalised to the set of biomarkers present-All). Table 1 presents the panel estimation of vNE generated by the fold-restricted entropy calculation on trainset. Significantly, the zero value of vNE in Panel C and Panel A is in line with high internal redundancy, whereas in Panel E, the multivariate dispersion is significantly large.

**Table 1:** Fold-isolated panel entropy (vNE) and normalized vNE (normalized to “All” when available). Values are extracted from the notebook output.

Group	vNE	vNE_norm
Panel_E	1.8892	2.4042
Panel_D	1.2590	1.6022
Panel_B	1.0712	1.3632
All	0.7858	1.0000
Panel_C	0.0045	0.0058
Panel_A	0.0004	0.0006

### T2. Bootstrapped panel-pair synergy shows limited and bounded uplift

In order to check the strength of apparent panel synergy (not leakage or full-dataset covariance), we checked panel pairs with bootstrapped synergy estimates. The most advanced pairs in terms of synergy are presented in Table 2 with the 95 bootstrap confidence intervals. Synergy ratios are moderate, and the confidence intervals do not indicate radical and unstable uplift which justifies the manuscript assertion that robust entropy-driven synergy cannot be maintained following leakage-safe processing.

**Table 2:** Bootstrapped synergy for selected panel pairs (extracted from notebook output). $S_{A\cup B}$ is the entropy of the combined panel; $S_{\text{boot}}$ is bootstrap mean synergy score with 95 % CI.

	$G_{B}$	$S_{A}$	$S_{B}$	$S_{A\cup B}$	$S_{\text{ratio}}$	$S_{\text{boot}}$	95 % CI
Panel_B	Panel_E	1.0712	1.8892	1.9131	0.6462	0.6576	[0.5803, 0.7724]
Panel_D	Panel_E	1.2590	1.8892	1.8902	0.6004	0.5988	[0.5581, 0.6313]
Panel_B	Panel_D	1.0712	1.2590	1.2376	0.5311	0.5226	[0.4002, 0.6233]

### T3. Entropy–prediction coupling: AUROC does not scale monotonically with vNE

Entropy magnitude alone does not guarantee predictive discrimination. We therefore paired each panel (and selected panel unions) with a consistent 5-fold cross-validated AUROC evaluation. Table 3 shows that Panel_B achieves AUROC comparable to the “All” biomarker set under the same evaluation protocol, while some higher-entropy panels do not yield higher AUROC. This validates the need for coupling entropy screening with predictive benchmarking in a single auditable workflow.

**Table 3:** Panel-level predictive benchmarking (5-fold CV AUROC) extracted from notebook output. “GBM” corresponds to gradient boosting; “MLP” corresponds to a multilayer perceptron baseline.

Group	AUROC (GBM)	AUROC (MLP)	#Features
All	0.7068	0.6316	36
All∪Panel_C	0.7068	0.6316	36
Panel_B	0.7067	0.6659	7
Panel_A∪Panel_C	0.7053	0.5403	14
Panel_C	0.6881	0.5482	8
Panel_A	0.6735	0.5305	6
Panel_E	0.6573	0.4552	8
Panel_D	0.4846	0.4598	7

The Clinical-Lifestyle grouping achieved AUROC = 0.973 under the same leakage-safe protocol. However, this grouping includes structured demographic and established risk-related variables rather than purely serum biomarker measurements. Therefore, its strong predictive discrimination should not be interpreted as an entropy-driven biomarker synergy but as reflecting a well-established epidemiological risk structure evaluated under the same leakage-controlled conditions.

### T4. Demonstration of leakage inflation in naive full-feature modeling

To justify the necessity of the leakage-audit and fold-isolated screening protocol, we evaluated multiple standard classifiers using the full numeric feature set without the panel-level leakage-safe restriction. This naive setting yields near-perfect AUROC for several models (Table 4),

**Table 4:** Naive full-feature baseline shows inflated discrimination (5-fold mean metrics) extracted from notebook output. This motivates leakage auditing and fold-isolated screening.

Model	AUROC	PR-AUC	Macro-F1	Brier
LogReg_L1	0.9999	0.9990	0.9792	0.0032
RandomForest	0.9999	0.9993	0.9876	0.0034
XGBoost	0.9999	0.9990	0.9918	0.0018
CatBoost	1.0000	1.0000	0.9917	0.0020
GaussianNB	0.9434	0.7056	0.6010	0.0710

Such near-perfect discrimination is statistically implausible for heterogeneous biomarker-based ovarian cancer risk modelling and strongly indicates the presence of administrative or post-outcome leakage pathways. The attenuation of AUROC from approximately 1.0 in naive full-feature modelling to approximately 0.70 under the leakage-aware panel protocol demonstrates the practical necessity of structured administrative auditing and fold-restricted entropy computation.

In contrast, the leakage-aware panel benchmarking (Table 3) yields AUROC ≈0.70, consistent with attenuated performance once leakage pathways are controlled.

### T5. Leakage keyword scan highlights administrative/follow-up candidates

As an auditable support step, a keyword-based scan flagged variables consistent with administrative/follow-up encoding (e.g., exit status, eligibility, outcome-days). Table 5 lists representative flagged fields (subset shown). These variables are treated as leakage candidates and excluded prior to entropy computation in the leakage-aware protocol.

**Table 5:** Representative leakage candidates flagged by a keyword-based scan (subset; extracted from notebook output).

Feature	Flagged as possible leakage
ovar_exitage	True
bq_compdays	True
entrydays_dhq	True
entrydays_sqx	True
ovar_eligible_dhq	True
ovar_eligible_sqx	True
ovar_eligible_bq	True
ovar_exitstat	True
sample_drawdays	True
mortality_exitstat	True

## Method implementation summary

Across these checks, the results support the central methodological claims: (1) entropy computed from full-dataset covariance can be contaminated by held-out statistics, (2) naive full-feature modeling produces inflated discrimination consistent with leakage, and (3) the proposed leakage-aware, fold-isolated, panel-level entropy screening coupled with predictive benchmarking yields attenuated and more credible performance estimates and bounded synergy behavior. Entropy estimates demonstrated low variance across cross-validation folds, supporting stability of fold-restricted covariance estimation. This suggests that the proposed protocol yields reproducible panel-level complexity measurements under stratified sampling.

## Generalizability of the workflow

Although the present demonstration uses PLCO ovarian cancer data, the workflow is not ovarian-specific. The protocol is applicable to any structured biomedical dataset in which variables can be grouped into biologically, clinically, or technically meaningful panels. This includes proteomic panels, radiomic feature blocks, transcriptomic pathways, multimodal clinic genomic groupings, and longitudinal clinical feature sets. The leakage audit step is dataset-agnostic, while the fold-restricted entropy step applies whenever multivariate structure is summarized through covariance-derived quantities. The predictive benchmarking component is likewise modular and can be implemented with alternative classifiers, provided that all fitting and tuning remain confined to the training folds.

## Comparison with existing leakage-aware feature selection practices

Standard feature selection methods such as mutual information ranking and minimum-redundancy maximum-relevance (mRMR) [7] aim to identify informative individual features while minimizing redundancy. When embedded properly within cross-validation, such approaches can mitigate feature-level leakage. However, they operate primarily at the individual-feature level and do not explicitly evaluate the covariance structure of predefined feature groups.

Regularization-based approaches such as LASSO [4] embed feature selection within predictive modeling but similarly do not quantify multivariate entropy of grouped variables. Nested cross-validation frameworks are widely recommended to prevent optimistic bias arising from model selection and hyperparameter tuning. While effective for general validation control, these frameworks are not specifically designed for entropy-based screening derived from covariance matrices. (Table 6)

**Table 6:** Comparison with existing leakage-aware feature-selection practice.

Approach	Primary Objective	Controls Feature-Level Leakage	Controls Panel-Level Covariance Leakage	Integrates Information Screening with Predictive Benchmarking
Mutual Information / mRMR [7]	Rank individual features by maximizing relevance and minimizing redundancy	Yes, when embedded within cross-validation	No; operates at individual-feature level	Typically no; screening often performed separately from final validation
Regularization-Based Selection (e.g., LASSO) [16]	Embedded feature selection within predictive modeling	Yes	No; does not explicitly evaluate grouped covariance structure	Implicitly through model performance
Nested Cross-Validation [14,17]	Prevent optimistic bias from model selection and hyperparameter tuning	Yes	Not specifically designed for entropy or covariance-based screening	Yes
Leakage Auditing Frameworks [12,13]	Identify outcome-proximal and administrative leakage pathways	Yes (conceptual and procedural guidance)	Not specific to entropy-based covariance estimation	Not inherently integrated with entropy screening
**Proposed Leakage-Aware Entropy Workflow (This Study)**	Structured panel-level entropy screening under fold-restricted validation	Yes (explicit administrative audit + fold-restricted preprocessing)	Yes; covariance matrices and entropy computed strictly within training folds	Yes; entropy screening directly coupled with cross-validated predictive benchmarking and calibration metrics

Leakage auditing literature emphasizes the risks posed by administrative or outcome-proximal variables and provides conceptual guidance for identifying such risks. However, these works do not explicitly address multivariate entropy estimation under fold isolation.

The present workflow extends leakage-aware validation principles to panel-level entropy screening by treating covariance estimation itself as a fold-restricted training operation. Entropy computation is never performed on the full dataset prior to validation. Additionally, entropy-based panel screening is directly coupled with cross-validated predictive benchmarking and calibration assessment, reducing the risk that apparent interaction patterns arise from subtle information leakage rather than genuine multivariate structure.

## Limitations

The method relies on accurate identification of administrative and post-outcome variables. If dataset documentation is incomplete, leakage detection may require domain expertise. Von Neumann entropy measures covariance structure and does not directly infer biological causality. High predictive performance of certain clinical groupings should be interpreted cautiously and externally validated.

## Ethics statements

This study is a secondary analysis of de-identified data from the PLCO Cancer Screening Trial accessed via NCI CDAS. No direct human subject interaction was involved.

## Declaration of competing interest

The authors declare that they have no known competing financial interests or personal relationships that could have appeared to influence the work reported in this paper

## Appendix Supplementary materials

Image, application 1
