# Supplementary material for: A leakage-aware entropy screening protocol for structured biomarker panel evaluation in ovarian cancer risk modelling
Source: MethodsX. 2026 Mar 21;16:103880. doi: 10.1016/j.mex.2026.103880 (PMC13049967; doi:10.1016/j.mex.2026.103880)
Supplement: Supplementary file 1 [file mmc1.pdf]

Supplementary Material

Leakage-Aware Quantum-Inspired Entropy Screening for Biomarker Panel Evaluation

S1. Leakage Audit and Variable Exclusion

S1.1 Motivation

During exploratory modeling, certain variables demonstrated disproportionately high predictive importance relative to canonical biomarkers. Manual inspection revealed that these variables were administrative or follow-up indicators, potentially encoding outcome information indirectly.

To ensure that entropy and predictive metrics reflect genuine multivariate structure rather than administrative encoding, a structured leakage audit was implemented.

S1.2 Exclusion Criteria

Variables were removed if they satisfied one or more of the following:

- Encoded exit status or case resolution.
- Contained mortality or follow-up endpoints.
- Represented eligibility or enrollment flags.
- Directly or indirectly derived from case-control labels.
- Reflected post-diagnosis administrative fields.

S1.3 Verification Procedure

After removal:

- Correlation between remaining features and outcome was re-evaluated.
- Feature importance distributions were recalculated.
- No single feature exhibited dominance suggestive of leakage.

All preprocessing and entropy computations were restricted to training folds only.

S2. Panel-Level Entropy and Predictive Metrics

Five-fold stratified cross-validation (random seed = 42) was applied to $n = 1101$ samples.

Table 1: Panel-level entropy and predictive performance (mean across folds)

Panel	AUROC	Accuracy	Macro-F1	Brier	ECE	vNE (mean)
Clinical_Lifestyle	0.973	0.956	0.871	0.034	0.016	0.644
Panel_B	0.705	0.911	0.681	0.079	0.046	1.071
All_Biomarkers	0.703	0.905	0.671	0.081	0.047	0.786
Panel_C	0.683	0.903	0.664	0.084	0.040	0.005
Reproductive	0.562	0.889	0.479	0.098	0.034	2.380

Interpretation

Clinical_Lifestyle demonstrated strong predictive discrimination under the same leakage-safe pipeline. Panel_C exhibited near-zero entropy, indicating strong internal redundancy. All_Biomarkers did not substantially outperform Panel_B alone, suggesting overlapping informational structure.

S3. Pairwise Panel Evaluation

Table 2: Selected pairwise panel combinations

Panel Pair	AUROC	vNE (mean)
B + C	0.720	0.0046
A + B	0.719	0.0005
B + E	0.708	1.913
A + C	0.699	0.782

Observations

- No pair demonstrated dramatic uplift relative to individual biomarker panels.
- Combinations involving Panel_B showed relatively stable performance.
- Entropy magnitude alone did not guarantee predictive improvement.

S4. Effect of Leakage Mitigation

In preliminary exploratory analysis conducted prior to leakage audit, certain panel combinations appeared to show modest predictive uplift.

After strict removal of administrative and post-outcome variables:

- Apparent synergy between Panel A and Panel C was attenuated.

- Predictive uplift reduced.
- Confidence intervals widened.
- Feature importance became more evenly distributed.

This confirms that entropy-derived conclusions are sensitive to leakage and must be validated under fold-restricted preprocessing.

S5. Entropy Computation Details

For each training fold:

$$C_j = \mathrm{Cov}(X_j) \tag{1}$$

$$\rho_j = \frac{C_j}{\mathrm{tr}(C_j)} \tag{2}$$

$$S(\rho_j) = -\sum_k \lambda_k \log_2 \lambda_k \tag{3}$$

Eigenvalues below 10^{-12} were discarded to ensure numerical stability.
Bootstrap resampling ($B = 1000$) was used to estimate entropy confidence intervals.

S6. Computational Environment

- Python 3.10
- NumPy
- SciPy
- Scikit-learn
- XGBoost
- Stratified 5-fold cross-validation
- Random seed: 42

Entropy computation, imputation, and scaling were restricted to training folds to prevent information leakage.

S7. Limitations of Entropy Screening

- Von Neumann entropy measures covariance structure and does not infer biological causality.
- High predictive discrimination of clinical groupings should be externally validated.
- Panel definitions were predefined and not optimized.

S8. Core Implementation Code (Reproducibility)

This section provides the main Python functions used in the leakage-aware entropy screening protocol. The snippets reflect the essential reproducibility logic and can be adapted to other datasets.

S8.1 Leakage-safe column removal

```
def drop_leakage_cols(df, leak_cols):
    """
    Remove administrative or post-outcome variables.
    """
    if leak_cols is None or len(leak_cols) == 0:
        return df
    return df.drop(columns=leak_cols, errors="ignore")
```

S8.2 Fold-wise preprocessing (train-only fitting)

```
from sklearn.impute import SimpleImputer
from sklearn.preprocessing import StandardScaler

def fit_preprocessors(X_train):
    imputer = SimpleImputer(strategy="median")
    scaler  = StandardScaler()

    Xtr_imp = imputer.fit_transform(X_train)
    Xtr     = scaler.fit_transform(Xtr_imp)
    return imputer, scaler

def apply_preprocessors(X, imputer, scaler):
    X_imp = imputer.transform(X)
    X_out = scaler.transform(X_imp)
    return X_out
```

S8.3 Von Neumann entropy computation

```
import numpy as np

def vne_from_data(X, eps=1e-12):
    """
    Compute von Neumann entropy from covariance-derived density matrix.
    """
    if X.shape[1] < 2:
        return 0.0
```

```
    C = np.cov(X, rowvar=False)
    C = 0.5 * (C + C.T)

    tr = np.trace(C)
    if tr <= eps:
        return 0.0

    rho = C / tr
    w = np.linalg.eigvalsh(rho)
    w = w[w > eps]

    if w.size == 0:
        return 0.0

    return float(-(w * np.log2(w)).sum())
```

S8.4 Bootstrap confidence intervals

```
import numpy as np

def bootstrap_ci(values, B=1000, alpha=0.05, seed=42):
    rng = np.random.default_rng(seed)
    values = np.asarray(values, dtype=float)
    n = len(values)

    if n == 0:
        return np.nan, np.nan, np.nan

    boots = []
    for _ in range(B):
        samp = rng.choice(values, size=n, replace=True)
        boots.append(np.mean(samp))

    boots = np.asarray(boots)
    lo = np.quantile(boots, alpha/2)
    hi = np.quantile(boots, 1 - alpha/2)

    return float(np.mean(values)), float(lo), float(hi)
```

S8.5 Performance metrics

```
from sklearn.metrics import roc_auc_score, f1_score
import numpy as np

def brier_score(y_true, y_prob):
```

```
    y_true = np.asarray(y_true).astype(float)
    y_prob = np.asarray(y_prob).astype(float)
    return float(np.mean((y_prob - y_true) ** 2))

def expected_calibration_error(y_true, y_prob, n_bins=10):
    y_true = np.asarray(y_true).astype(int)
    y_prob = np.asarray(y_prob).astype(float)

    bins = np.linspace(0.0, 1.0, n_bins + 1)
    ece = 0.0
    n = len(y_true)

    for i in range(n_bins):
        lo, hi = bins[i], bins[i+1]
        if i < n_bins - 1:
            mask = (y_prob >= lo) & (y_prob < hi)
        else:
            mask = (y_prob >= lo) & (y_prob <= hi)

        if not np.any(mask):
            continue

        acc = np.mean(y_true[mask] == (y_prob[mask] >= 0.5))
        conf = np.mean(y_prob[mask])
        ece += (np.sum(mask) / n) * abs(acc - conf)

    return float(ece)
```

S8.6 Leakage-safe cross-validation loop

```
from sklearn.model_selection import StratifiedKFold
from xgboost import XGBClassifier

def eval_panel_cv(X, y, leak_cols=None, n_splits=5, seed=42):
    skf = StratifiedKFold(n_splits=n_splits, shuffle=True, random_state=seed)

    aucs, f1s, briers, eces = [], [], [], []

    for tr_idx, te_idx in skf.split(X, y):
        Xtr, Xte = X.iloc[tr_idx].copy(), X.iloc[te_idx].copy()
        ytr, yte = y.iloc[tr_idx], y.iloc[te_idx]

        Xtr = drop_leakage_cols(Xtr, leak_cols)
        Xte = drop_leakage_cols(Xte, leak_cols)
```

```
        imputer, scaler = fit_preprocessors(Xtr)
        Xtr_p = apply_preprocessors(Xtr, imputer, scaler)
        Xte_p = apply_preprocessors(Xte, imputer, scaler)

        model = XGBClassifier(
            n_estimators=400,
            max_depth=3,
            learning_rate=0.05,
            subsample=0.9,
            colsample_bytree=0.9,
            random_state=seed,
            eval_metric="logloss"
        )

        model.fit(Xtr_p, ytr)
        p = model.predict_proba(Xte_p)[:, 1]

        aucs.append(roc_auc_score(yte, p))
        f1s.append(f1_score(yte, (p >= 0.5).astype(int), average="macro"))
        briers.append(brier_score(yte, p))
        eces.append(expected_calibration_error(yte, p))

    return {
        "auroc": np.mean(aucs),
        "macro_f1": np.mean(f1s),
        "brier": np.mean(briers),
        "ece": np.mean(eces)
    }
```

S8.7 Fold-wise entropy estimation

```
def entropy_cv(X, y, leak_cols=None, n_splits=5, seed=42):
    skf = StratifiedKFold(n_splits=n_splits, shuffle=True, random_state=seed)
    entropies = []

    for tr_idx, _ in skf.split(X, y):
        Xtr = X.iloc[tr_idx].copy()
        Xtr = drop_leakage_cols(Xtr, leak_cols)

        imputer, scaler = fit_preprocessors(Xtr)
        Xtr_p = apply_preprocessors(Xtr, imputer, scaler)

        entropies.append(vne_from_data(Xtr_p))

    mean_e, lo, hi = bootstrap_ci(entropies, B=1000, seed=seed)
```

```
    return {"vne_mean": mean_e, "vne_lo": lo, "vne_hi": hi}
```

S8.8 Composite ranking of panels and pairs

```
import pandas as pd

def add_composite_ranks(df):
    df = df.copy()

    df["rank_auroc"] = df["auroc"].rank(ascending=False, method="min")
    df["rank_macro_f1"] = df["macro_f1"].rank(ascending=False, method="min")
    df["rank_brier"] = df["brier"].rank(ascending=True, method="min")
    df["rank_ece"] = df["ece"].rank(ascending=True, method="min")

    df["rank_sum"] = (
        df["rank_auroc"] +
        df["rank_macro_f1"] +
        df["rank_brier"] +
        df["rank_ece"]
    )

    df["rank_overall"] = df["rank_sum"].rank(ascending=True, method="min")
    return df
```

S8.9 Utility for generating panel combinations

```
from itertools import combinations

def build_panel_pairs(panel_dict):
    """
    panel_dict: {panel_name: [feature_list]}
    Returns dictionary of pair_name -> combined feature list
    """
    pair_dict = {}
    for (name1, feats1), (name2, feats2) in combinations(panel_dict.items(), 2):
        pair_name = f"{name1}+{name2}"
        pair_dict[pair_name] = list(set(feats1 + feats2))
    return pair_dict
```

S9. Supplementary Conclusion

The supplementary analyses provide complete transparency regarding leakage mitigation, entropy computation, panel evaluation, and composite ranking procedures used in the main manuscript.

The results confirm that, after strict removal of administrative and post-outcome variables and implementation of fold-restricted preprocessing, previously observed exploratory synergy patterns were attenuated. No biomarker pair demonstrated strong and consistent entropy-driven predictive uplift under leakage-safe validation.

The inclusion of Clinical-Lifestyle variables under the same leakage-controlled framework demonstrates that high predictive discrimination can be preserved when appropriate safeguards are applied.

All entropy estimates, confidence intervals, cross-validation metrics, and ranking procedures are reproducible using the provided code segments. The supplementary material therefore substantiates the methodological robustness of the leakage-aware entropy screening protocol.

The intent of this supplementary documentation is to ensure that panel-level entropy screening can be independently replicated, audited, and adapted to other biomedical datasets without reliance on implicit preprocessing assumptions.
